# Intraindividual Behavioral Variability Predicts Foraging Outcome in a Beach-dwelling Jumping Spider

**DOI:** 10.1038/s41598-017-18359-x

**Published:** 2017-12-22

**Authors:** James L. L. Lichtenstein, Gregory T. Chism, Ambika Kamath, Jonathan N. Pruitt

**Affiliations:** 1 Department of Ecology, Evolution and Marine Biology, University of California Santa Barbara, Santa Barbara, CA 93106 USA; 2 Graduate Interdisciplinary Program in Entomology and Insect Science, University of Arizona, Tucson, AZ 85721 USA

## Abstract

Animal personality, defined as consistent differences between individuals in behavior, has been the subject of hundreds if not thousands of papers. However, little work explores the fitness consequences of variation in behavior within individuals, or intraindividual variability (IIV). We probe the effects of behavioral IIV on predator-prey interaction outcomes in beach-dwelling jumping spiders (*Terralonus californicus*). Prior studies have found that spiders with higher body condition (body mass relative to size) behave more variably. Thus, we hypothesized that jumping spider activity level IIV would relate positively to foraging performance. To address this, we tested for associations between activity IIV, average activity level, and two measures of foraging success in laboratory mesocosms: change in spider mass and the number of prey killed. Activity IIV positively correlated with the mass that spiders gained from prey, but not with the number of prey killed. This suggests that spiders with high IIV consumed a greater proportion of their prey or used less energy. Interestingly, average activity level (personality) predicted neither metric of foraging success, indicating that behavioral IIV can predict metrics of success that personality does not. Therefore, our findings suggest that IIV should be considered alongside personality in studies of predator-prey interactions.

## Introduction

Animal personality refers to temporally consistent individual differences in behavior^1^. To assess these differences, behavioral ecologists run individual animals through multiple iterations of various behavioral tests, and then take the average of each individual’s performance on these tests to assign them to a specific personality or behavioral type. Much personality research aims to parse the causes and consequences of these individual behavioral averages^2–5^. For instance, individual averages in traits like activity level and boldness can predict mating success, predator success, and prey survival rates^6–11^. However, individuals also differ in their variability or consistency in behaviors like boldness or activity level^12^. For instance, in hermit crabs, one individual may take risks consistently over its lifespan, while another may be daring one day and then cautious the next^12–14^. This behavioral intraindividual variability (IIV) is defined as within-individual variability in performance on a given behavioral test, corrected for systematic variation arising from habituation or fatigue^12,15,16^. Yet, unlike individual behavioral averages^2,3,6^, the ecological and evolutionary consequences of behavioral IIV remain unclear^12,17,18^.

This opacity stems in part from conflicting evidence on the fitness consequences of behavioral IIV. Across taxa, behavioral IIV can correspond with high success^19^ or low success^20^. For example, birds that sing variable and inconsistent songs have lower mating success^21–23^, and risk-taking IIV in fish correlates with lower foraging performance^20^. These studies suggest that behavioral variability reflects the lack of a cohesive behavioral strategy, perhaps akin to confusion or panic, resulting in poor performance and fitness. Conversely, other researchers have reasoned that high behavioral IIV and unpredictability in general could be adaptive^13,24,25^. Unpredictable movement by spiders, sometimes termed “protean behavior”, can render spiders less susceptible to being captured by *Portia* spiders^19,26,27^, and more behaviorally variable spiders have been found to be more likely to win contests with conspecifics^28^. Behavioral inconsistency (IIV) can be beneficial or costly depending on whether it reflects adaptive plasticity or maladaptive confusion. The benefits of variability have been examined in relation to mating success and prey survival, but never predator success.

Therefore, we tested whether behavioral inconsistency (IIV), alongside average behavior (personality) and body size, predicts the outcome of predator-prey interactions between the beach jumping spider, *Terralonus californicus* (Araneae, Salticidae), and the beach fly, *Fucellia rufitibia*. We chose these species, because we know that *T*. *californicus* eats *F*. *rufitibia* based on extensive feeding trials and carbon isotope analyses (McLaughlin & Chism, unpublished data), and because spiders are one of the most voracious predator groups on earth, consuming 400–800 million tons of prey a year, rivaling humans and whales^29^. Spiders have also been fruitful model organisms for the study of personality and behavioral IIV^30^. Studies on widow spiders (*Latrodectus hesperus*) have found that individuals exhibit greater behavioral IIV when they have been raised on high quality diets and are in good body condition^31,32^. This and other studies suggest that behavioral IIV is associated with high body condition and performance in spiders^28,32,34^. Based on this association between IIV and high body condition, we hypothesize that behaviorally inconsistent (high IIV) spiders are more effective predators.

Thus, we first attempted to confirm whether activity level IIV was associated with spider body condition. Body condition is often used as a proxy for fitness, and we quantify body condition as the residual distance of spider mass from a regression of body mass on carapace width^33^. Subsequently, we tested whether spiders with high activity level IIV ate more prey and gained more mass. We chose activity level, measured as the distance that spiders moved in five minutes in an open field test, because it characterizes the aggressive stalking hunting mode of jumping spiders^9,35^. We also tested whether average activity level and body size (prosoma width) were associated with predator success. We first calculated average activity level and activity level IIV in 25 *T*. *californicus* from five test iterations. Next, the spiders went through week-long mesocosm trials where they were free to consume prey in small plastic enclosures, with 12 prey items per enclosure. We evaluated the ability of spiders’ behavioral variability (IIV) to predict the outcome of this predator-prey interaction, as to clarify the ecological consequences of intraspecific behavioral variation in *T*. *californicus*.

## Results

Spiders were significantly repeatable in their activity level (R = 0.449, CI_lower_ = 0.370, CI_upper_ = 0.598). To screen for potential collinearity in our models we tested for correlations between activity level IIV, average activity level, and prosoma width. Prosoma width was not significantly correlated with either average activity level (R^2^ = 0.002, L-R Chi^2^ = 0.051, p = 0.821; Supplementary Figure S1a) or activity level IIV (R^2^ = 0.049, L-R Chi^2^ = 1.663, p = 0.280; Supplementary Figure S1b). Activity level IIV was not correlated with average activity level (R^2^ = 0.049, L-R Chi^2^ = 0.205, p = 0.651; Supplementary Figure S1c).

### Behavioral IIV and body condition

Activity level IIV was unrelated to pre-trial body condition (R^2^ = 0.043, L-R Chi^2^ = 1.013, p = 0.314; Supplementary Figure S2a) and post-mesocosm body condition (n = 25, R^2^ = 0.134, L-R Chi^2^ = 3.319, p = 0.685; Supplementary Figure S2b).

### Predator-prey interaction outcome

**Figure 1:**
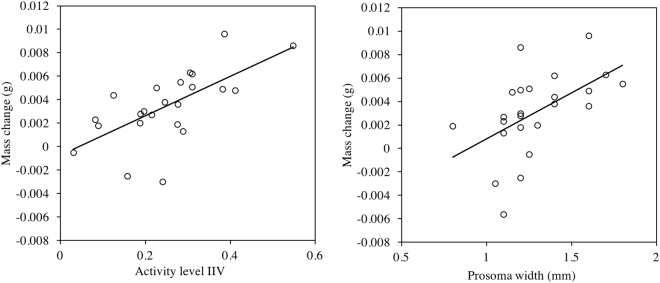
Change in spider mass is correlated with (**a**) Activity level IIV (GLM effect test: n = 25, L-R Chi^2^ = 6.158, p = 0.013) and (**b**) Prosoma width (GLM effect test: n = 25, L-R Chi^2^ = 7.110 p = 0.008). Activity level refers to performance on an open field test, and IIV refers to inter-individual variation estimated by riSD. Lines represent linear best fit. Spider activity level intraindividual variability (IIV) (**a**) change in body condition (GLM effect test: n = 25, L-R Chi^2^ = 6.158 p = 0.013) but not (**b**) number of flies eaten (GLM effect test: L-R Chi^2^ = 1.102 p = 0.294). Activity level refers to performance on an open field test, body condition refers to the residual definition of body condition (residual distance from predicted mass)^33^. Trend lines represent best fit regressions.

We tested whether spider behavioral traits predict the outcome of their interaction with flies, specifically the number of kelp flies eaten and the mass gained from week-long mesocosm trials. Spiders underwent a 63% absolute change in their mass on average by the end of the mesocosm trials. The whole model, containing activity level IIV, average activity level, and prosoma width as fixed effects, significantly predicted mass change (Table 1). Specifically, spider activity level IIV and prosoma width were related to mass change (Table 1; Fig. 1), whereas average activity level and prosoma width had no effect on mass change (Table 1). To confirm these findings, we ran identical analyses on change in body condition. Activity level IIV (GLM effect test: n = 25, L-R Chi^2^ = 12.309 p = 0.0005; Supplementary Figure S3a) and prosoma width (GLM effect test n = 25, L-R Chi^2^ = 9.384, p = 0.002; Supplementary Figure S3b) had positive effects on spider body condition gain.

**Table 1:** Outputs of models comparing aspects of spider behavior to metrics of predator-prey interaction outcome. The * refers to results that are significant at the α = 0.05 level. The Activity IIV and Average Activity rows refer to effect tests. Activity level refers to performance on an open field test, body condition refers to the residual definition of body condition (residual distance from predicted mass)^33^.

Response variable	Predictor variable	R^2^	df	L-R Chi^2^	p
Change in mass	Whole model	0.586	3.000	20.309	*0.0001
	Activity level IIV		1.000	12.309	*0.005
	Average activity level		1.000	0.553	0.457
	Prosoma width		1.000	6.984	*0.008
Flies eaten	Whole model	0.447	3.000	8.772	*0.032
	Activity level IIV		1.000	1.102	0.294
	Average activity level		1.000	2.201	0.138
	Prosoma width		1.000	4.320	*0.038

**Figure 2:**
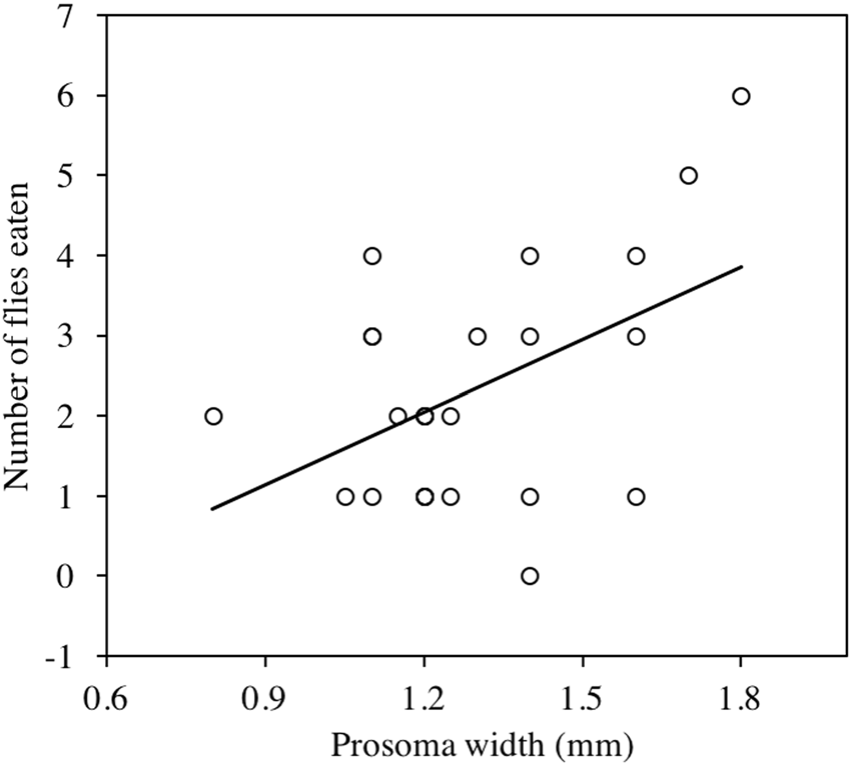
Prosoma width (mm) predicts the number of flies eaten (GLM effect test: n = 25, L-R Chi^2^ = 4.320 p = 0.038). Trend line represent best fit regression.

The whole model containing activity level IIV, average activity level, and prosoma width had a significant effect on the number of flies consumed (Table 1). This is because spiders with greater prosoma width consumed more flies (Table 1; Fig. 2). Interestingly, the number of flies consumed was not significantly correlated with change in body condition (R^2^ = 0.101, L-R Chi^2^ = 2.545, p = 0.111).

## Discussion

To probe how behavioral inconsistency (IIV) relates to predator-prey interactions, we tested whether jumping spider activity level IIV was related to low^20^ or high^31^ predator mass gain and predator-prey interaction outcome. We found that spider IIV was unrelated to pre- and post-mesocosm body condition. This suggests that the variation in IIV that we observed is unrelated to the initial nutritional state of the spiders, conflicting with work on other spider species, which found that spiders in better body condition were more behaviorally variable^32,36^. Next, spiders with higher variability in activity levels increased more in mass and body condition (Fig. 1; Supplementary Figure S3), consistent with evidence suggesting that behavioral IIV in spiders is related to higher performance^28,31,37^.

Interestingly, average activity level predicted no metrics of predator success. This suggests that behavioral inconsistency (IIV) can predict outcomes of predator-prey interactions that average behavior (personality) cannot. This suggests that numerous studies probing the role of personality in spider feeding behavior^38–40^ may not have considered all relevant intraspecific behavioral variation. Notably, neither average activity level nor activity IIV predicted the number of prey that spiders consumed (Table 1), meaning that variation in activity level both within and across individuals is seemingly unrelated to prey capture success. Rather, larger spiders tended to catch more prey and gain more mass, and behaviorally variable spiders gained more mass without necessarily killing more prey (Figs 1 and 2). The effect of prosoma width on mass gain could be explained by the increased ability of large spiders to produce more digestive fluids. The increased mass gain of behaviorally variable spiders adds to growing evidence suggesting that behavioral IIV plays a role in predator-prey interactions^19,20^, but that the mechanism underlying its effects may be indirect. We discuss two possible explanations for the enhanced performance of behaviorally inconsistent (high IIV) spiders.

First, behaviorally consistent (low IIV) spiders could have gained less mass because they had faster metabolic rates. Spiders with greater metabolic rates would burn though their existing nutritional reserves more quickly, reducing their mass. Considerable evidence suggests that personality traits like aggressiveness, boldness, and activity level can be correlated with metabolic rate^5,41–43^. The same is potentially true for behavioral IIV. However, we found no relationship between spider IIV and pre-mesocosm body condition following an *ad libitum* feeding event (Supplementary Figure S2a). This result is inconsistent with a relationship between IIV and metabolic rate. Had individuals varied in metabolic rate as function of their activity level IIV, we would have predicted that individuals with a higher metabolic rate (low IIV) would have exhibited a lower body condition at a set time following an equivalent (*ad libitum*) feeding event. Based on this indirect evidence, we cannot definitively conclude whether or not metabolism is related to IIV in these spiders, but these data do raise some skepticism towards the possibility IIV is strongly related to metabolic rate. Factors such as nutritional history^31,44^, partial/differential prey consumption^45,46^, age/experience^47,48^, and hormonal state^49,50^ can also affect foraging decisions in spiders. These factors are beyond the scope of the present study, and at present confound our ability to more critically evaluate this hypothesis that IIV is related to metabolic rate.

Second, it is possible that behaviorally variable spiders consumed a greater proportion of the prey that they killed than behaviorally consistent spiders. Killing prey without fully consuming them is termed variably as superfluous killing, wasteful killing, or partial prey consumption, and occurs when predators kill more or larger prey than they can or will consume^51^. In the grass spider *Agelenopsis aperta*, and the comb-footed spider, *Anelosimus studiosus*, tendency to engage in superfluous killing or partial prey consumption varies consistently among individuals in the context of a broad boldness-aggressiveness behavioral syndrome^46,52^. We propose that a similar association between individual behavioral tendencies (here activity level IIV) and tendency to partially consume may occur in *T*. *californicus*. Partial or incomplete prey consumption can be adaptive or costly, depending on the situation. Partial prey consumption can reduce handling time and digestive fluid investment, because digested prey grow more viscous and difficult to ingest as they are consumed^45,53^. If behavioral IIV correlates with high juvenile nutrition and spider fitness^8,28^, then behaviorally inconsistent individuals be able to invest more in the production of digestive fluid, thus allowing them to gain more nutrients from their prey. Conversely, low IIV spiders may invest less in digestive fluid, and only consume choice prey portions before seeking out more prey.

The data herein add to growing evidence^54–56^ that intraspecific behavioral variation can predict individual performance in staged mesocosm. An interesting next step might be to probe the fitness consequences of behavioral IIV in unpredictable environments, or to manipulate juvenile environment to observe the cascading consequences it has on adult IIV. Most studies relating behavioral variation to foraging performance ignore IIV in favor of focusing on behavioral averages (behavioral types/personality). For instance, many have found that spider average activity level and aggressiveness predict the number and species of prey animals they consume^9,57,58^. Had we done this, we would have mistakenly concluded that intraspecific behavioral variation was unrelated to predator performance. However, evaluating IIV is more time consuming, and requires far more data than evaluating average behaviors, which may help to explain why few studies aim to address its effects. Nonetheless, the available evidence suggests that IIV can have sizable and contrasting effects compared to average behavior, and may enhance the predictability of species interaction modules^19,20^. Therefore, under ideal circumstance, both personality and behavioral IIV should be accounted for when probing the causes and consequences of intraspecific behavioral variation.

## Methods

### Subject species and collection

We collected *T*. *californicus* from sandy beach habitat adjacent to the University of California, Santa Barbara (34.406290°N, 119.847680°W) from August to October 2016. Individuals were collected with an inhalation aspirator apparatus (Bioquip 1135 A, 1135Y), and then transported to the University of California Santa Barbara, where they were stored individually in 12 ml (12 cm × 3 cm) plaster containers (Bioquip 8912) containing approximately 10 g of kelp (*Macrocystis pyrifera*) wrack. We selected only female spiders from a size range of 0.8 mm to 1.5 mm to ensure that they could subdue the prey we provided. They weighed on average 8.4 ± 4.4 mg SD (Supplementary Figure 4a). We collected specimens of the kelp fly *Fucellia rufitibia* (identified by GC) from beaches surrounding UCSB campus. We caught these phototactic flies by illuminating containers at night on kelp wracks and closing the containers when approximately 50 flies had entered. We chose this species pair because we know that *T*. *californicus* consumes *F*. *rufitibia in situ* based on extensive feeding trials, stable isotope analyses, and natural observations (McLaughlin & Chism, unpublished data).

### Activity level assays

We began spider behavioral testing four days after collection. Immediately after the spiders arrived in the lab, we fed them all to satiation with five *Bledius fenyesi* (Staphylinidae), a beetle species that these spiders prey upon in the wild. We began open field assays forty-eight hours after this feeding event.

Activity assays took place in a 120 mm × 120 mm square petri-dish with 1 cm grid paper fastened below it. We gave the spiders 30 seconds to acclimate to the dish before we started the trial. We then counted the number of squares the spiders crossed with their cephalothoraxes during the following 300 seconds. We chose the open field test for two reasons. First, it is a well vetted metric of activity level related to fitness and predatory behavior in numerous other species^59–61^. Second, this test is neither left nor right censored, which can bias behavioral IIV estimates^12^. The distribution of spiders’ performances on this test does not differ significantly from a normal distribution (Shapiro-Wilk W test: W = 0.99, p = 0.16). We performed this assay every other day for ten days (5 trials per spider).

### Predation experiment

After the activity level assays, we gave all spiders a 3-day rest period. We next measured the number of prey they killed and the change in their mass and body condition in a staged mesocosm experiment. We used two chambered mesocosms to create habitat complexity for the spiders and to allow them, and their prey, to move between habitat patches/refuges. The two-chamber setup mimics the natural habitat of these spiders, which is characterized by a series of discrete patches (clumps of decaying kelp) that the spiders move between. We constructed mesocosms using two 750 ml deli containers that we connected by a 15 cm section of 25 mm plastic tubing, and sealed the joints with silicone. We put 250 mg of sieved beach sand (using a 2 mm sieve) in each chamber, along with 30 ml of filtered water and ~10 g of cleaned kelp wrack to each patch (obtained fresh from the surf).

Before we put spiders into the mesocosms, we weighed each spider to the 10^−4^ gram (Denver Instruments Pinnacle balance) and measured their prosoma widths to the nearest millimeter (Bioquip 4828 M mini-scale). Next, we placed spiders in freshly provisioned mesocosms along with 12 prey items - *Fucellia rufitibia* (Anthomyiidae, Fucellidae). Twelve flies were more than a single spider could eat but still within the range of densities at which these flies occur *in situ* (Chism & Lichtenstein, pers. obs.). We left the mesocosms at 18 °C for six days exposed to natural light. Once the trial was completed, we recorded how many flies were killed by predators. Flies killed by predators were easily distinguishable from other deaths, because the corpses exhibited clear bite and feeding point marks, a lack of internal organs, or were mangled from predator handling. To determine changes in body condition, we reweighed and measured the prosoma widths of spiders immediately following the trial. We found no evidence that the spiders had molted during the trials. Thus, we recorded three metrics of predator success: change in body condition, the number of flies consumed, and change in mass. Spiders were then released within 10 m of their original collection site.

### Statistical methods

To assess IIV in activity level, we calculated the residual individual standard deviation (riSD) of each spider. The riSD approach is the most widely accepted estimation of IIV, because it accounts for habituation and other systematic sources of performance variation across days^12,14,19,62^. It is analogous to a standard deviation calculation, but instead of measuring deviation from the average, riSD is calculated with deviation from the expected performance for each day^12,63,64^. To calculate activity level riSD, we first made a linear model for each spider with “test day” as a predictor variable and “activity level” as a response variable. For each day, we used the estimated slope and intercept of this model to calculate an expected value. Next, we subtracted the expected value from the observed value, and then divided this by the expected value to get average corrected deviation (residuals) of their activity level scores from the expected value for each day. Activity level IIV is the average corrected residual for each spider, and the average IIV was 0.249 ± 0.115 SD (Supplementary Figure S4d) for our spiders. We calculated these values using the lm function in R version 3.3.1. Then, to test whether activity level was significantly repeatable we fit a GLMM with “spider ID’ as a random effect, “testing day” as a fixed effect, and activity level as a response variable. We assessed whether activity level was significantly repeatable by observing whether the confidence intervals of the spider ID random effect overlapped zero, after Nakagawa and Schielzeth^65^. We fit this model using the rptR package^66^ in R version 3.3.1.

We estimated the body condition of our spiders as the residuals of a regression of mass against prosoma width^33^. The residual distance method characterizes body condition as the deviation of spider’s mass from their expected mass for their given body size (prosoma width). We fit two GLMs with normal distributions, prosoma width as the predictor variable and spider mass as the response variable for before and after experiment values. Prosoma width was correlated with mass before (n = 25, R^2^ = 0.283, L-R Chi^2^ = 7.978, p = 0.047) and after (n = 25, R^2^ = 0.637, L-R Chi^2^ = 24.309, p > 0.0001) the experiment (Supplementary Figure S5). The residuals of these models conformed to normal distributions, implying that Gaussian distributions are good fits for the data. This metric of body condition was not correlated with prosoma width before (n = 25, R^2^ < 0.001, L-R Chi^2^ = 0.011, p = 0.917) but it was after the experiment (n = 25, R^2^ = 0.292, L-R Chi^2^ = 8.279, p = 0.004). We calculated pre-mesocosm body condition as the residual distance of spiders’ pre-mesocosm mass from the pre-mesocosm prosoma/mass regression and post-mesocosm body condition as the distance of their post-mesocosm mass from this same regression.

Next, to assess the effects of spider behavior on predator success, we used a series of GLMs. We fit two GLMs with normal distributions, with activity level IIV, average activity level, and prosoma width as predictor variables. One model had mass change as the response variable, and the other had change in body condition as the response variable. We fit a similar model for the number of flies consumed with average activity, activity IIV, and prosoma width as predictor variables, but instead fit the data with a GLM using a Poisson distribution. Once again, the residuals of all of these models conformed to normal distributions. To ensure that multicollinearity did not compromise these models, we tested to see whether activity level IIV, average activity level, and prosoma width were correlated using three linear models. We performed all statistics, excepting riSD and repeatability calculations, with JMP 13.0 Pro.

## Electronic supplementary material

Supplementary Figures S1–S4

## References

[1] Gosling SD (2001). From mice to men: what can we learn about personality from animal research?. Psychological bulletin.

[2] Sih A, Cote J, Evans M, Fogarty S, Pruitt J (2012). Ecological implications of behavioural syndromes. Ecology Letters.

[3] Wolf M, Weissing FJ (2012). Animal personalities: consequences for ecology and evolution. Trends in Ecology & Evolution.

[4] Bergmüller R, Taborsky M (2010). Animal personality due to social niche specialisation. Trends in Ecology & Evolution.

[5] Careau V, Thomas D, Humphries M, Réale D (2008). Energy metabolism and animal personality. Oikos.

[6] Smith BR, Blumstein DT (2008). Fitness consequences of personality: a meta-analysis. Behavioral Ecology.

[7] Pruitt JN, Stachowicz JJ, Sih A (2012). Behavioral types of predator and prey jointly determine prey survival: potential implications for the maintenance of within-species behavioral variation. The American Naturalist.

[8] DiRienzo N, Pruitt JN, Hedrick AV (2013). The combined behavioural tendencies of predator and prey mediate the outcome of their interaction. Animal Behaviour.

[9] Sweeney K (2013). Predator and prey activity levels jointly influence the outcome of long-term foraging bouts. Behavioral Ecology.

[10] Réale D, Martin J, Coltman D, Poissant J, Festa‐Bianchet M (2009). Male personality, life‐history strategies and reproductive success in a promiscuous mammal. Journal of evolutionary biology.

[11] Schuett W, Dall SR, Royle NJ (2011). Pairs of zebra finches with similar ‘personalities’ make better parents. Animal Behaviour.

[12] Stamps JA, Briffa M, Biro PA (2012). Unpredictable animals: individual differences in intraindividual variability (IIV). Animal Behaviour.

[13] Briffa M (2013). Plastic proteans: reduced predictability in the face of predation risk in hermit crabs. Biology letters.

[14] Briffa M, Bridger D, Biro PA (2013). How does temperature affect behaviour? Multilevel analysis of plasticity, personality and predictability in hermit crabs. Animal Behaviour.

[15] Carter AJ, Marshall HH, Heinsohn R, Cowlishaw G (2012). How not to measure boldness: novel object and antipredator responses are not the same in wild baboons. Animal Behaviour.

[16] Biro PA, Adriaenssens B (2013). Predictability as a personality trait: consistent differences in intraindividual behavioral variation. The American Naturalist.

[17] Jeanson R, Weidenmüller A (2014). Interindividual variability in social insects–proximate causes and ultimate consequences. Biological Reviews.

[18] Jandt JM (2014). Behavioural syndromes and social insects: personality at multiple levels. Biological Reviews.

[19] Chang, C.-c., Teo, H. Y., Norma-Rashid, Y. & Li, D. Predator personality and prey behavioural predictability jointly determine foraging performance. *Scientific Reports* **7** (2017). 10.1038/srep40734 PMC5240143 28094288

[20] Ioannou, C. C. & Dall, S. R. Individuals that are consistent in risk-taking benefit during collective foraging. *Scientific reports* **6** (2016). 10.1038/srep33991 PMC5037426 27671145

[21] Byers BE (2007). Extrapair paternity in chestnut-sided warblers is correlated with consistent vocal performance. Behavioral Ecology.

[22] Botero CA (2009). Syllable type consistency is related to age, social status and reproductive success in the tropical mockingbird. Animal Behaviour.

[23] Węgrzyn E, Leniowski K, Osiejuk TS (2010). Whistle duration and consistency reflect philopatry and harem size in great reed warblers. Animal Behaviour.

[24] Bednekoff PA, Lima SL (2002). Why are scanning patterns so variable? An overlooked question in the study of anti‐predator vigilance. Journal of Avian Biology.

[25] Humphries D, Driver P (1967). Erratic display as a device against predators. Science.

[26] Jones, K. A., Jackson, A. L. & Ruxton, G. D. Prey jitters; protean behaviour in grouped prey. *Behavioral Ecology*, arr062 (2011).

[27] Humphries D, Driver P (1970). Protean defence by prey animals. Oecologia.

[28] Riechert SE (1978). Games spiders play: behavioral variability in territorial disputes. Behavioral Ecology and Sociobiology.

[29] Nyffeler M, Birkhofer K (2017). An estimated 400–800 million tons of prey are annually killed by the global spider community. The Science of Nature.

[30] Pruitt JN, Riechert SE (2012). The ecological consequences of temperament in spiders. Current Zoology.

[31] DiRienzo N, Montiglio PO (2016). The contribution of developmental experience vs. condition to life history, trait variation and individual differences. Journal of Animal Ecology.

[32] Pruitt JN, DiRienzo N, Kralj-Fišer S, Johnson JC, Sih A (2011). Individual-and condition-dependent effects on habitat choice and choosiness. Behavioral Ecology and Sociobiology.

[33] Jakob, E. M., Marshall, S. D. & Uetz, G. W. Estimating fitness: a comparison of body condition indices. *Oikos*, 61–67 (1996).

[34] Araya‐Ajoy, Y. G. & Dingemanse, N. J. Repeatability, heritability, and age‐dependence in the aggressiveness reaction norms of a wild passerine bird. *Journal of Animal Ecology* (2016). 10.1111/1365-2656.12621 27973682

[35] Miller JR, Ament JM, Schmitz OJ (2014). Fear on the move: predator hunting mode predicts variation in prey mortality and plasticity in prey spatial response. Journal of Animal Ecology.

[36] Wright CM, Keiser CN, Pruitt JN (2015). Personality and morphology shape task participation, collective foraging and escape behaviour in the social spider Stegodyphus dumicola. Animal Behaviour.

[37] Lichtenstein JL (2016). Prolonged food restriction decreases body condition and reduces repeatability in personality traits in web-building spiders. Behavioral Ecology and Sociobiology.

[38] Pruitt, J. N. *et al*. The Achilles’ heel hypothesis: misinformed keystone individuals impair collective learning and reduce group success. *Proc*. *R*. *Soc*. *B*. 20152888 (2016). 10.1098/rspb.2015.2888 PMC4795039 26817771

[39] Keiser, C. N. & Pruitt, J. N. Spider aggressiveness determines the bidirectional consequences of host–inquiline interactions. *Behavioral Ecology*, art096 (2013).

[40] Wright CM (2017). Exposure to predators reduces collective foraging aggressiveness and eliminates its relationship with colony personality composition. Behavioral Ecology and Sociobiology.

[41] Lantová P, Zub K, Koskela E, Šíchová K, Borowski Z (2011). Is there a linkage between metabolism and personality in small mammals? The root vole (Microtus oeconomus) example. Physiology & behavior.

[42] Lichtenstein J, Pruitt J (2015). Similar patterns of frequency‐dependent selection on animal personalities emerge in three species of social spiders. Journal of evolutionary biology.

[43] Lichtenstein, J. L. *et al*. Participation in cooperative prey capture and the benefits gained from it are associated with individual personality. *Current Zoology*, zow097 (2016). 10.1093/cz/zow097 PMC5637736 29033979

[44] Mayntz D, Raubenheimer D, Salomon M, Toft S, Simpson SJ (2005). Nutrient-specific foraging in invertebrate predators. Science.

[45] Riechert, S. E. & Maupin, J. L. Spider effects on prey: tests for superfluous killing in five web-builders. *Proceedings of the 17th European Colloquium of Arachnology*, *Edinburgh*. 203–210 (1998).

[46] Maupin JL, Riechert SE (2001). Superfluous killing in spiders: a consequence of adaptation to food-limited environments?. Behavioral Ecology.

[47] Rayor LS, Uetz GW (1993). Ontogenetic shifts within the selfish herd: predation risk and foraging trade-offs change with age in colonial web-building spiders. Oecologia.

[48] Persons MH, Uetz GW (1999). Age and sex-based differences in the use of prey sensory cues in wolf spiders (Araneae: Lycosidae). Journal of insect behavior.

[49] Gaskett A (2007). Spider sex pheromones: emission, reception, structures, and functions. Biological Reviews.

[50] Trabalon M, Niogret J, Legrand-Frossi C (2005). Effect of 20-hydroxyecdysone on cannibalism, sexual behavior, and contact sex pheromone in the solitary female spider, Tegenaria atrica. General and comparative endocrinology.

[51] Conover RJ (1966). Factors affecting the assimilation of organic matter by zooplankton and the question of superfluous feeding. Limnology and Oceanography.

[52] Pruitt JN, Riechert SE, Jones TC (2008). Behavioural syndromes and their fitness consequences in a socially polymorphic spider, Anelosimus studiosus. Animal Behaviour.

[53] Samu F (1993). Wolf spider feeding strategies: optimality of prey consumption in Pardosa hortensis. Oecologia.

[54] Lichtenstein, J. L., Pruitt, J. N. & Modlmeier, A. P. Intraspecific variation in collective behaviors drives interspecific contests in acorn ants. *Behavioral Ecology*, arv188 (2015).

[55] Toscano BJ, Griffen BD (2014). Trait‐mediated functional responses: predator behavioural type mediates prey consumption. Journal of Animal Ecology.

[56] Pruitt JN (2017). Behavioral Hypervolumes of Predator Groups and Predator-Predator Interactions Shape Prey Survival Rates and Selection on Prey Behavior. The American Naturalist.

[57] Royauté R, Pruitt JN (2015). Varying predator personalities generates contrasting prey communities in an agroecosystem. Ecology.

[58] Pruitt JN, Ferrari MC (2011). Intraspecific trait variants determine the nature of interspecific interactions in a habitat‐forming species. Ecology.

[59] Carter AJ, Feeney WE, Marshall HH, Cowlishaw G, Heinsohn R (2013). Animal personality: what are behavioural ecologists measuring?. Biological Reviews.

[60] Dingemanse NJ, Both C, Drent PJ, Van Oers K, Van Noordwijk AJ (2002). Repeatability and heritability of exploratory behaviour in great tits from the wild. Animal behaviour.

[61] Kurvers RH (2009). Personality differences explain leadership in barnacle geese. Animal Behaviour.

[62] Jennings DJ, Hayden TJ, Gammell MP (2013). Personality and predictability in fallow deer fighting behaviour: the relationship with mating success. Animal Behaviour.

[63] Hultsch DF, Strauss E, Hunter MA, MacDonald SW (2008). Intraindividual variability, cognition, and aging. The handbook of aging and cognition.

[64] Hoffman L (2007). Multilevel models for examining individual differences in within-person variation and covariation over time. Multivariate Behavioral Research.

[65] Nakagawa S, Schielzeth H (2010). Repeatability for Gaussian and non‐Gaussian data: a practical guide for biologists. Biological Reviews.

[66] Nakagawa S, Schielzeth H (2013). A general and simple method for obtaining R2 from generalized linear mixed‐effects models. Methods in Ecology and Evolution.

