# Supplementary material for: Intraindividual Behavioral Variability Predicts Foraging Outcome in a Beach-dwelling Jumping Spider
Source: Sci Rep. 2017 Dec 22;7:18063. doi: 10.1038/s41598-017-18359-x (PMC5741732; doi:10.1038/s41598-017-18359-x)
Supplement: Supplementary file 1 — Supplementary Figures S1–S4 [file 41598_2017_18359_MOESM1_ESM.doc]

**intraindividual behavioral variability predicts foraging outcome in a beach-dwelling jumping spider**

James L.L. Lichtenstein†*^1^, Gregory T. Chism†^2^, Ambika Kamath^1^, Jonathan N. Pruitt^1^

^1^ Department of Ecology, Evolution and Marine Biology, University of California Santa Barbara, Santa Barbara, CA 93106

^2^ Graduate Interdisciplinary Program in Entomology and Insect Science, University of Arizona, Tucson, AZ 85721

† denotes equal contributions

* denotes corresponding author


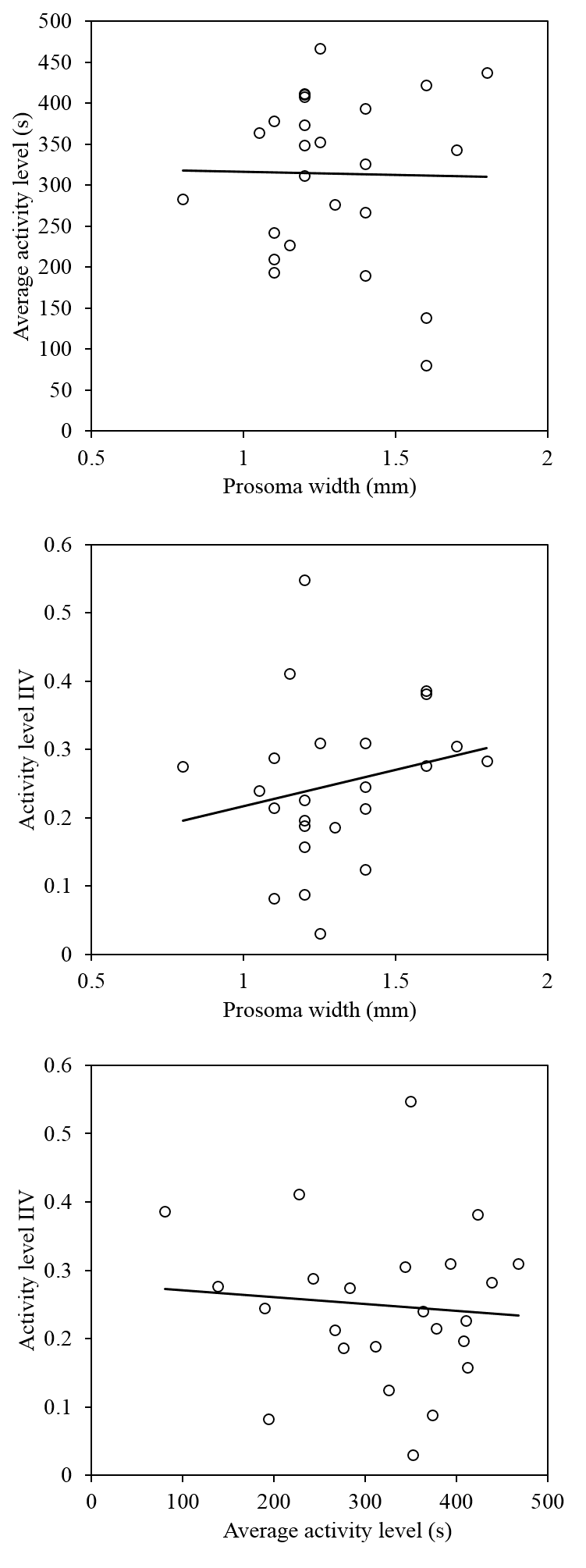


Supplementary Figure S1. There are no relationships between Prosoma width, (a) average activity level (n = 25, R^2^ = 0.002, L-R Chi^2^ = 0.051, p = 0.821) and (b) activity level intraindividual variation (IIV) (n = 25, R^2^ = 0.049, L-R Chi^2^ = 1.663, p = 0.280), and (c) average activity level and prosoma width (n = 25, R^2^ = 0.049, L-R Chi^2^ = 0.205, p = 0.651). Activity level refers to performance on an open field test, and IIV refers to inter-individual variation estimated by riSD. Lines represent linear best fit.


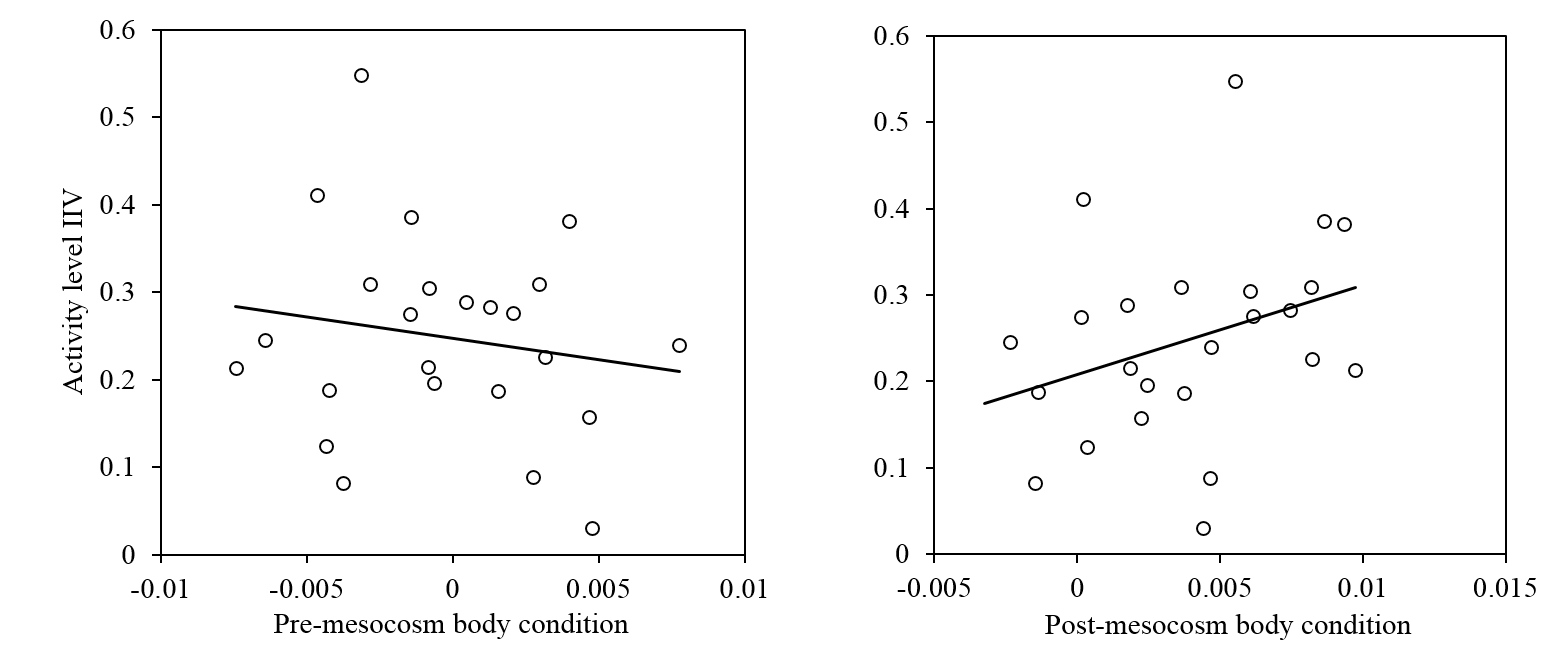


Supplementary Figure S2. There is no relationship between activity level intraindividual variation (IIV) and body condititon (a) before (n = 25, R^2^ = 0.043, L-R Chi^2^ = 1.013, p = 0.314) and (b) after (n = 25, R^2^ = 0.134, L-R Chi^2^ = 3.319, p = 0.069) the mesocosm experiment. Activity level refers to performance on an open field test, body condition refers to the residual definition of body condition (residual distance from predicted mass). Line represents linear best fit.


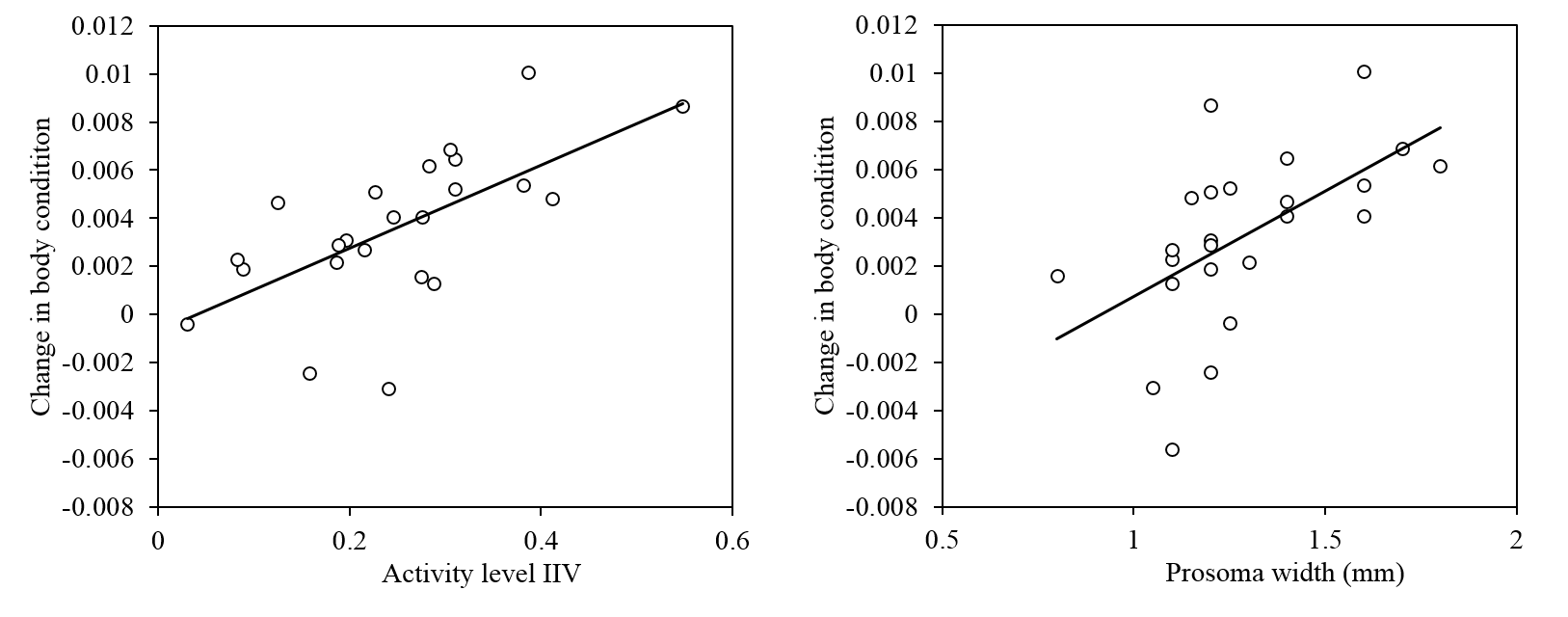


Supplementary Figure S3. Spider activity level intraindividual variability (IIV) (a; GLM effect test: n = 25, L-R Chi^2^ = 12.309 p = 0.0005) and prosoma width (b; GLM effect test n = 25, L-R Chi^2^ = 9.384, p = 0.002) correlate with change in body condition. Activity level refers to performance on an open field test, body condition refers to the residual definition of body condition (residual distance from predicted mass)^33^. Trend lines represent best fit regressions.


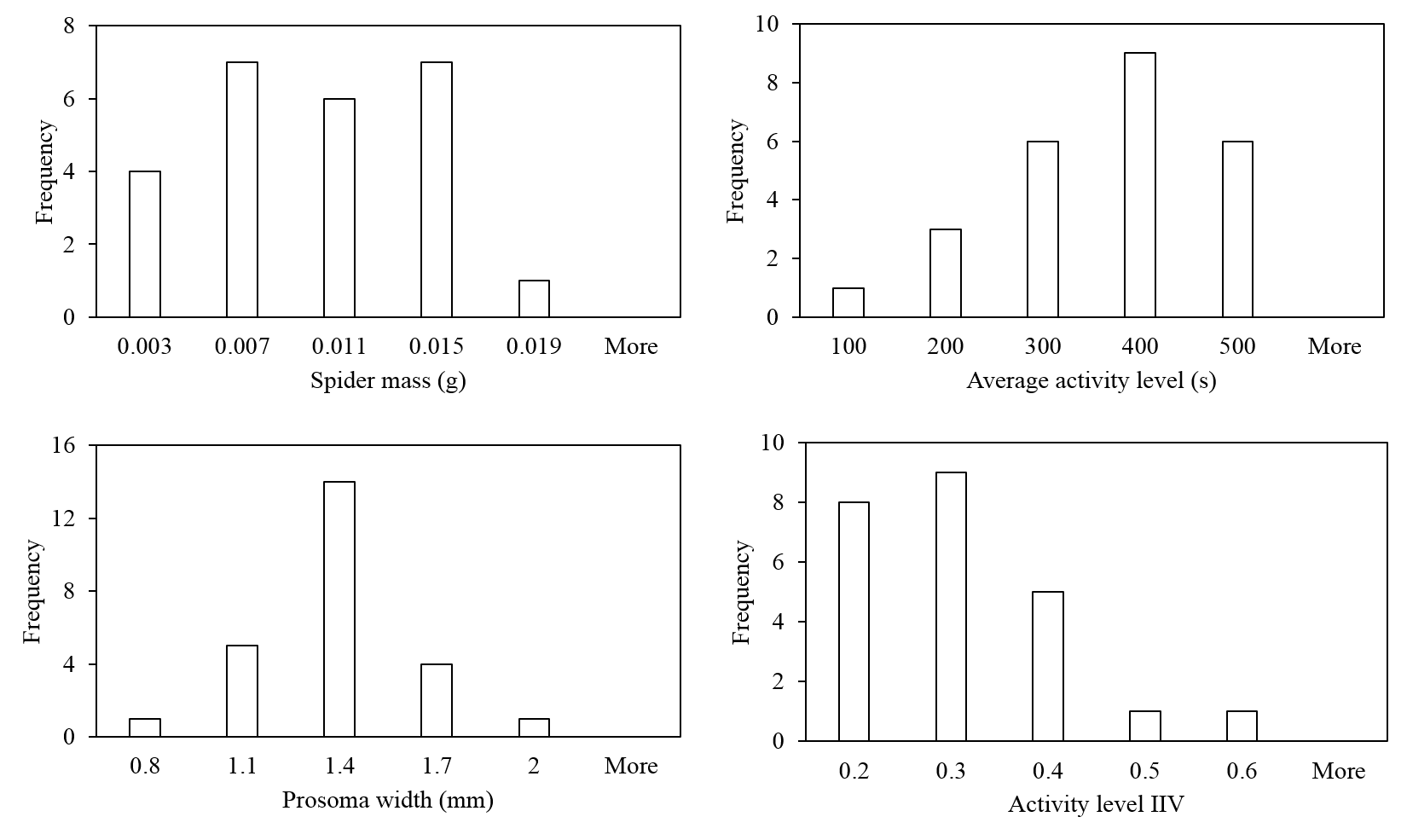


Supplementary Figure S4. Histograms of (a) spider mass, (b) average activity level, (c) prosoma width, and (d) Activity level IIV. Activity level refers to performance on an open field test, and IIV refers to inter-individual variation estimated by riSD.

**
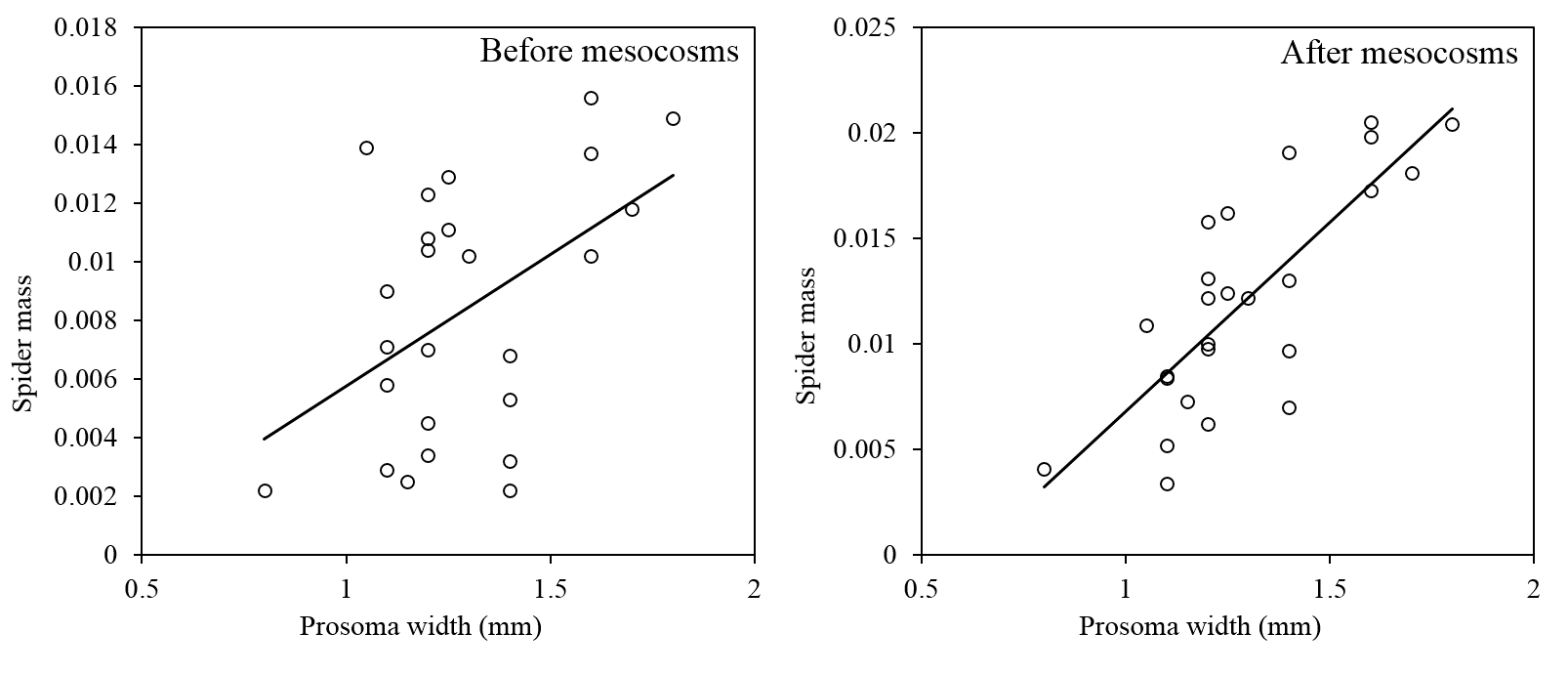
**

Supplementary figure S5. Spider prosoma width correlates with spider mass before (a; n = 25, R^2^ = 0.283, L-R Chi^2^ = 7.978, p = 0.047) and after the mesocosm experiment (b; n = 25, R^2^ = 0.637, L-R Chi^2^ = 24.309, p > 0.0001). Trend lines represent best fit regressions.
